# Factors influencing the success of patch therapy in patients with intermittent exotropia: a retrospective case-control study

**DOI:** 10.1186/s12886-026-04774-0

**Published:** 2026-04-11

**Authors:** Jee Hyun Jeong, Se Youp Lee, Dong Cheol Lee

**Affiliations:** Department of Ophthalmology, Keimyung University School of Medicine, 1095 Dalgubeol-daero, Dalseo-gu, Daegu, 42601 Republic of Korea

**Keywords:** Intermittent exotropia, Patch therapy, Strabismus

## Abstract

**Background:**

Part-time occlusion is used to delay or avoid surgery in intermittent exotropia; however, the treatment criteria and optimal duration remain unclear. This retrospective case-control study aimed to identify factors influencing deviation angle normalization and recurrence risk after patch therapy in intermittent exotropia.

**Methods:**

Patients with intermittent exotropia who underwent 2–4-hours patching were divided into treatment success and failure groups; the treatment success group was divided into recurring and non-recurring subgroups. Patients whose deviation angle recovered to 10 prism diopters (PD) or under were seen as treatment success, patients whose deviation angle went 15 PD or more in the 1 year follow up after treatment success was achieved were seen as recurring group. Visual acuity, refraction, deviation angle, control, stereoacuity, patching time, and duration were investigated. The main outcomes included characteristics of patients whose deviation angle normalized. The independent t-test and chi-square test were used to compare continuous and categorical variables, respectively. Shapiro-Wilk normality testing was conducted before analyses. Cox regression analysis was used to compare the treatment success and treatment failure groups. Logistic regression analysis was used to compare the recurring and non-recurring subgroups within the treatment success group. Kaplan–Meier survival analysis was performed to calculate the probability of treatment success, and the log-rank test was used to compare the patching duration between the treatment failure and success groups.

**Results:**

Among the 59 patients included, 30 required surgeries due to treatment failure. Eleven of 29 patients in the treatment success group experienced recurrence. The deviation angle normalized within an average of 494 days in successfully treated patients; it normalized within 331 days in 50% of them. The treatment failure group had a larger initial deviation angle (*p* ≤ .0001). Shorter patching duration was a predictive factor for treatment success (*p* = .002). However, no significant factors were found between recurring and non-recurring cases.

**Conclusions:**

Patch duration and good compliance, especially considering age, are key factors in treatment success.

**Supplementary Information:**

The online version contains supplementary material available at 10.1186/s12886-026-04774-0.

## Background

Intermittent exotropia (IXT) is characterized by intermittent outward eye deviation and is the most common form of strabismus in the Asian population [1–4]. IXT has been classified into four types: basic, divergence excess, convergence insufficiency, and pseudo-divergence excess [5]. The primary goal of IXT treatment is to correct misalignment of the eyes, maintain or restore binocularity, and ensure normal vision development. Various surgical strategies, such as recession and resection of the rectus muscle, and non-surgical interventions, such as orthoptic vision therapy, overcorrecting minus lens therapy, occlusion therapy, and prism therapy, are available [6]; however, the effectiveness of these interventions in permanent alleviation of deviation remains unclear.

Part-time occlusion therapy, also known as patching of the dominant eye or alternative patching is considered an effective conservative management strategy to delay or avoid surgery. It has been shown to reduce scotoma suppression, increase fusion, and improve deviation control [7–11]. Patching has also been associated with a decrease in the magnitude of deviation, an increase in surgical success rates, and maintenance or improvement of stereopsis [8, 12, 13].

Nevertheless, complete improvement without surgical treatment is rare. To the best of our knowledge, only one study by Choi et al. [14] has demonstrated significant improvement with patch therapy alone. In the above mentioned prospective study, a reduction in deviation angle and improvement in deviation control and stereoacuity were observed in 31.6% of patients after one year of patch therapy. Researchers have also suggested that patients with large-angle deviations and poor distance stereopsis show significant improvement with occlusion therapy [14]. However, there is a lack of evidence regarding the indications for treatment and the optimal duration of treatment. Therefore, this study aimed to compare the characteristics in patients with IXT who underwent part-time patching therapy, recovered to prism diopters (PD) of 10 or under, experienced recurrence, or ultimately required surgical intervention. Additionally, the study aimed to identify factors that may be associated with the success of patch therapy.

## Methods

This retrospective case-control study analyzed the clinical records from Keimyung University Dongsan Hospital’s electronic medical record system of 277 patients under 10 years of age, diagnosed with IXT between May 2015 and December 2021. We aimed to compare the characteristics in patients with IXT who underwent part-time patching therapy, returned to a normal deviation angle range, experienced recurrence, or ultimately required surgical intervention. Additionally, the study aimed to identify factors that may be associated with the success of patching therapy. This study was approved by the Institutional Review Board (IRB) of Keimyung University Dongsan Hospital (approval no. 2022-02-031). This study adhered to the Strengthening the Reporting of Observational Studies in Epidemiology (STROBE) reporting guidelines and tenets of the Declaration of Helsinki. Owing to the retrospective nature of this study, the requirement for informed consent was waived.

Only patients with basic IXT whose distance deviation is within 10 PD of the near deviation and who underwent patch therapy were included in this study [6]. Moreover, patients had to have a duration of patch therapy of at least 3 months and follow-up of at least 1 year. Patients with the following conditions were excluded: (i) convergence insufficiency, divergence excess, or A-V pattern strabismus; (ii) previous treatment for exotropia; (iii) amblyopia, anisometropia, paralytic strabismus, high myopia (≥ -6.0 D), high hyperopia ( ≥ + 4.0 D); or (iv) other coexisting ocular pathologies. About excluding amblyopia and anisometropia patients, these conditions could be a result from strabismus but can happen vice versa. These patients can also benefit from patching but what this study tried to find out was more focused on patching treatment affecting exotropia, not amblyopia.

We obtained data on patient deviation, control, stereoacuity before treatment, duration of patch therapy (months between start and end of patching), hours of patching per day, uncorrected visual acuity using ETDRS vision test charts, and spherical equivalent refraction (SER) values. The angle of deviation in PD at a distance (4 m) and near (33 cm) was measured using the alternate cover test. The Titmus stereotest (Stereo Optical Co., Chicago, IL) was used to measure stereoacuity at 40 cm. Deviation control was classified as good, fair, or poor using a 3-point scale at both near (40 cm) and at a distance (4 m) proposed by Rosenbaum et al. [15], where ‘good control’ represented breaks only after cover testing with resuming fusion rapidly without blinking or refixating, ‘fair control’ represented breaks after fusion disrupted by cover testing with resuming fixation after blinking or refixation, and ‘poor control’ represented breaks spontaneously without disruption of fusion.

Patch therapy was applied to the dominant eye for 2–4 h based on the severity of the deviation selected by the physician for each patient, without a specific protocol. Alternate patching was applied when no definite dominant eye was detected [16]. Treatment success was defined as deviation ≤ 10 PD after initiation of patch treatment, while recurrence was defined as deviation ≥ 15 PD throughout the follow up 1 year after treatment success was achieved. After determining treatment success, patching was tapered to 2 h per day prior to cessation, as performed in a previous study on patients with amblyopia [17].

The primary analysis compared the characteristics in patients with treatment success or failure who underwent surgery. Additional analyses were performed in patients who experienced successful treatment outcomes and the recurring and non-recurring cases within this group.

### Statistical analyses

All statistical analyses were performed using SPSS version 23.0 (IBM, Armonk, NY). Visual acuity was transformed to the minimum angle of resolution (logMAR), while stereoacuity was transformed to log arc/s values. The independent t-test was conducted for continuous variables, and the chi-squared test was used for categorical variables to compare different groups. Normality testing was conducted before analysis. Cox regression analysis was used to compare patients with treatment success and treatment failure. Logistic regression analysis was used to compare between recurring and non-recurring cases among the treatment-success patients. Kaplan–Meier survival analysis was used to calculate the probability of treatment success based on the duration of patching and for IXT recurrence. The log-rank test was used to compare the duration of patching between the two groups divided according to the median value of each parameter. Statistical significance was set at *P* < .05.

## Results

A total of 59 (male: female, 35:24) patients were included in the study. We observed treatment success in 29 patients. Of these, recurrence occurred in 13 patients. Meanwhile, 30 patients did not respond to treatment and ultimately required surgery. The mean age was 5.79 ± 2.90 (1–11) years in the treatment success group and 7.17 ± 2.61 (1–11) years in the surgery group. There was no significant difference noted (*P* = .0612). A significantly larger angle of deviation at initial presentation was observed at both far and near distances in the surgery group than in the treatment success group (*P* < .0001, *P* = .0001) (Table 1). In the treatment success group, the angle of deviation after treatment was 4.63 ± 4.05 (0–10) PD and 6.88 ± 4.32 (0–14) PD at far and near distances, respectively. Among the non-recurring patients, the angle of deviation after treatment was 5.23 ± 3.70 (0–10) PD and 6.15 ± 3.87 (0–14) PD at far and near distances, respectively. Among the recurring patients, the angle of deviation at the time of recurrence was 19.39 ± 2.40 (16–25) PD and 20.23 ± 4.68 (16–30) PD at far and near distances, respectively.

**Table 1:** Demographic and clinical features of the treatment success and treatment failure groups^a^

	Success (*n* = 29)	Failure (*n* = 30)	*P*-value
Age (years)	5.79 ± 2.90 (1–11)	7.17 ± 2.61 (1–11)	0.06^†^
Sex			0.52^*^
Female / Male	13 (44.83) / 16 (55.17)	11 (36.67) / 19 (63.33)	
VA_OD	0.61 ± 0.28 (0.125–1)	0.72 ± 0.22 (0.4–1)	0.13^†^
VA_OS	0.68 ± 0.26 (0.2–1)	0.73 ± 0.22 (0.4–1)	0.42^†^
LogMAR_OD	0.27 ± 0.26 (0–0.9)	0.17 ± 0.17 (0–0.4)	0.10^†^
LogMAR_OS	0.21 ± 0.21 (0–0.7)	0.16 ± 0.13 (0–0.4)	0.29^†^
SER, D_OD	-0.50 ± 1.39 (-3.25 - +1.5)	-0.60 ± 1.08 (-4.50 - +0.75)	0.77^†^
SER, D_OS	-0.33 ± 1.30 (-2.93 - +2.0)	-0.56 ± 1.25 (-5.375 - +0.75)	0.49^†^
Angle_Far (PD)	18.07 ± 4.96 (12–30)	23.57 ± 4.37 (16–32)	< 0.0001^†^
Angle_Near (PD)	19.93 ± 5.23 (12–30)	25.50 ± 5.18 (18–37)	0.0001^†^
Control_Far			0.76^*^
Good/Fair/Poor	3 (18.75)/6 (37.5)/7 (43.75)	4 (14.81)/8 (29.63)/15 (55.56)	
Control_Near			0.48^*^
Good/Fair/Poor	2 (12.5)/7 (43.75)/7 (43.75)	5 (18.52)/7 (25.93)/15 (55.56)	
Log Arcsec	1.86 ± 0.52 (1.3–3.48)	1.99 ± 0.46 (1.6–2.6)	0.51^†^
Patch			0.53
OD/OS/Alternative	8 (27.59)/8 (27.59)/13 (44.83)	12 (41.38)/7 (24.14)/10 (34.48)	
Patch_Time/day (h)	2.97 ± 1.02 (1–4)	3.00 ± 0.96 (2–4)	0.90^†^
Patch_Duration (months)	11.97 ± 10.59 (4–74)	14.90 ± 15.34 (3–42)	0.40^†^

Abbreviations: OD, oculus dexter; OS, oculus sinister; PD, prism diopter; SER, spherical equivalent refraction

^a^Values are presented as number (%) or means ± standard deviations

^*^Chi-square test, ^†^independent t-test

Log Arcsec values were measured in near distance

**Fig. 1:**
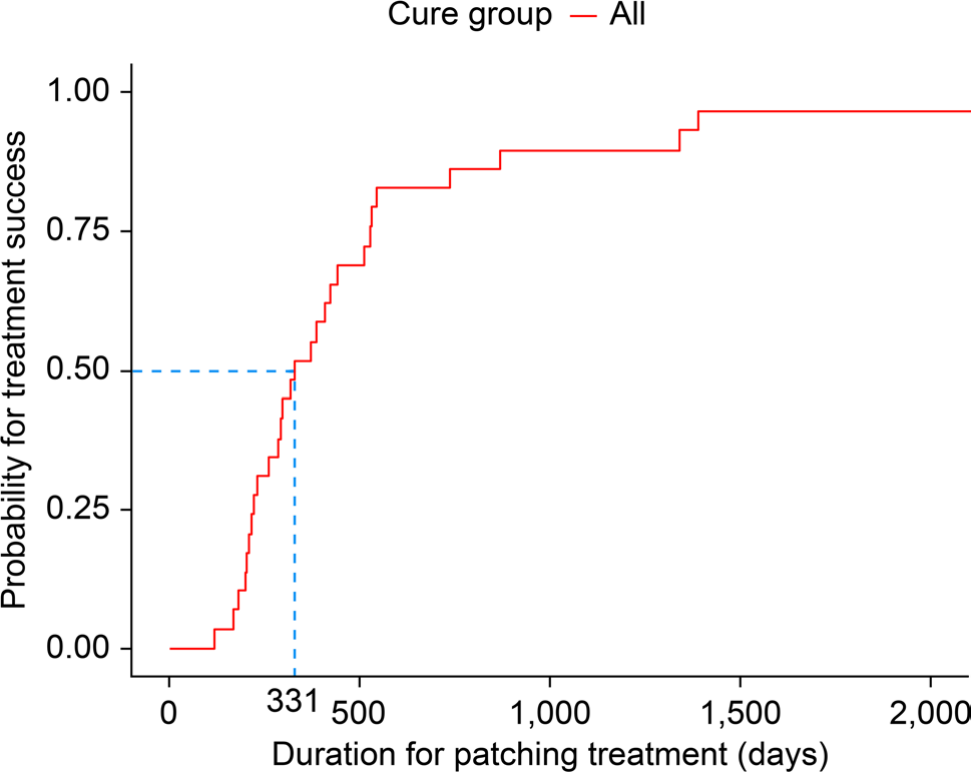
Kaplan–Meier survival plot for the rate of patch therapy success. The arrow indicates the follow-up period (331 days) for a 50% success rate after the start of treatment. Treatment success is observed after a mean of 494 days following a return to the normal deviation angle

Univariate analysis revealed the following factors as contributors to treatment success: older age (hazard ratio [HR], 1.25; 95% confidence interval [CI], 1.05–1.50; *P* = .01), smaller angle of deviation at distance (HR, 0.92; 9 5% CI, 0.85–0.99; *P* = .03), better deviation control at a distance (fair to good; HR, 0.21; 95% CI, 0.05–0.94; *P* = .04, poor to good; HR, 0.19; 95% CI, 0.04–0.84; *P* = .03), and shorter patching duration (HR, 0.77; 95% CI, 0.69–0.85; *P* < .0001). Of these, only patching duration was determined to be significant in multivariate analysis (HR, 0.73; 95% CI, 0.60–0.89; *P* = .002; Table 2). Kaplan–Meier analysis revealed a successful patching duration of 11.03 months (331 days) for 50% of the treatment success group (Fig. 1).

**Table 2:** Cox regression analysis of predictors of treatment success

		Univariate			Multivariate^a^		
		HR	CI	*P*-Value	HR	CI	*P*-value
Sex	Male	ref.					
	Female	1.16	(0.53, 2.51)	0.71			
Age		1.25	(1.05, 1.50)	0.01	1.18	(0.80, 1.75)	0.41
Angle_Far		0.92	(0.85, 0.99)	0.03	0.90	(0.76, 1.06)	0.20
Angle_Near		0.94	(0.87, 1.02)	0.15			
LogMAR OD		1.07	(0.13, 8.91)	0.95			
LogMAR OS		0.30	(0.03, 3.23)	0.32			
SER_OD		0.77	(0.55, 1.07)	0.12			
SER_OS		0.80	(0.58, 1.09)	0.16			
Control_Far	Good	ref.					
	Fair	0.21	(0.05, 0.94)	0.04	1.50	(0.15, 14.98)	0.73
	Poor	0.19	(0.04, 0.84)	0.03	0.55	(0.06, 4.66)	0.58
Control_Near	Good	ref.					
	Fair	0.44	(0.08, 2.32)	0.33			
	Poor	0.33	(0.06, 1.78)	0.20			
Log Arcsec		0.94	(0.29, 3.00)	0.91			
Patch_Duration/ day (h)		0.77	(0.69, 0.85)	< 0.0001	0.73	(0.60, 0.89)	0.002
Patch_Time (months)		0.69	(0.46, 1.04)	0.07			

Abbreviations: HR, hazard ratio; CI, confidence interval; OD, oculus dexter; OS, oculus sinister; SER, spherical equivalent refraction

^a^Model adjusted for age, deviation angle (far), deviation control, and patching duration

In the treatment success group, a longer patching duration time was associated with patients aged < 7 years (mean 19.84 months days, *P* = .07), a distance deviation angle of > 18 PD (mean 17.46 months, *P* = .97), near deviation angle of ≤ 20 PD (mean 18.84 months, *P* = .40), logMAR of > 0.2 in the right eye (mean 17.29 months, *P* = .69) and > 0.15 in the left eye (mean 23.98 months, *P* = .005), the spherical equivalent of > -0.5625 in the right eye (mean 20.76 months, *P* = .24) and > -0.1875 in the left eye (mean 20.68 months, *P* = .26), log arcsec of ≤ 1.70 (mean 13.95 months, *P* = .01), and lower deviation control both at a distance and near (mean 22.25 months in poor, *P* = .31 and 0.65, respectively). However, only logMAR of the left eye (*P* = .005) and log arcsec (*P* = .01) showed significant differences (Table 3).

**Table 3:** Comparison of the patching duration between the two groups divided according to the median value of each parameter using the log-rank test^a^

		Mean	Standard error	50% estimate	*P*-value
Total	Success	16.47	2.85	11.03	-
Age	< 7 years	19.84	4.33	14.47	0.07
	≥ 7 years	10.96	1.65	10.73	
Angle_Far	≤ 18 PD	15.54	2.78	11.03	0.97
	> 18 PD	17.46	5.2	11.22	
Angle_Near	≤ 20 PD	18.84	4.35	10.88	0.40
	> 20 PD	12.6	2.17	12.53	
LogMAR_OD	≤ 0.2	16.04	4.71	10.35	0.69
	> 0.2	17.29	4.55	12.53	
LogMAR_OS	≤ 0.15	9.71	1.31	8.35	0.005
	> 0.15	23.98	6.14	17.17	
SER_OD	≤ -0.5625	12.35	1.94	10.88	0.24
	> -0.5625	20.76	5.44	12.32	
SER_OS	≤ -0.1875	12.43	1.91	10.88	0.26
	> -0.1875	20.68	5.45	12.32	
Log Arcsec	≤ 1.70	13.95	2.13	12.9	0.01
	> 1.70	8.76	0.86	8.6	
Control_Far	Good	10.13	1.8	10.73	0.31
	Fair	21.86	7.53	11.88	
	Poor	22.25	8.94	14.83	
Control_Near	Good	11.82	1.08	11.82	0.65
	Fair	19.7	6.72	96.67	
	Poor	22.25	8.94	14.83	

Abbreviations: OD, oculus dexter; OS, oculus sinister; SER, spherical equivalent refraction

^a^Patients in the treatment success group alone are included

^b^Duration value is marked in months

**Fig. 2:**
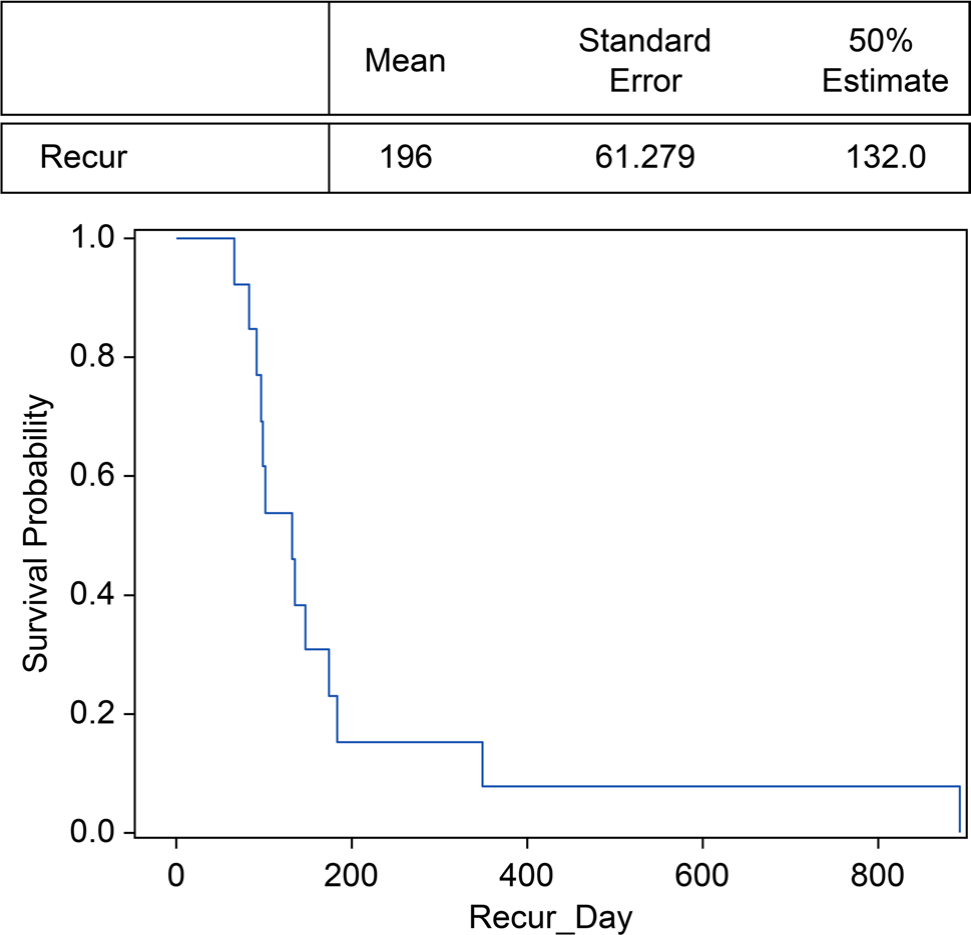
Kaplan–Meier survival plot for recurrence after patch therapy. Recurrence occurs after a mean of 196 days after achieving a normal deviation range

The demographic characteristics of the patients in the treatment success non-recurring and recurring groups are presented in the Supplementary Table S1. We found that patients in the non-recurring group were older (mean age: 6.75 ± 3.07 years) than those in the recurring group (mean age: 4.62 ± 2.29 years) (*P* = .05). Logistic regression analysis revealed no statistically significant differences between the non-recurring and recurring groups (Supplementary Table S2). In addition, recurrence occurred after a mean of 6.53 months (196 days) after the normal deviation range was observed (Fig. 2).

## Discussion

Patching is the preferred option for conservative management of IXT due to its lack of serious adverse effects and ease of accessibility. While many studies have reported the effectiveness of patch therapy [8, 9, 12, 13, 18, 19], the specific factors that contribute to treatment success or recurrence have not yet been well established.

In this study, older age, smaller deviation angle at distance, good deviation control at distance, and shorter patching duration were identified as influential factors for treatment success in the univariate analysis. After adjusting for these four variables, a shorter patching duration was found to be significant (*P* = .002). Each variable was then divided into two categories and compared for the duration of patching. We found that applying patch therapy over a longer treatment duration period was associated with better stereoacuity. No variables were found to be significantly associated with recurrence after successful treatment.

According to these results, shorter patching duration was a predictive factor for treatment success. This finding can be interpreted in two ways. First, patching has been shown to decrease the angle of deviation and improve the success rate after surgery [12]. Therefore, in this study, patients in the treatment failure group continued treatment until surgery was performed, meaning the treatment failure group had a longer patching period regardless of treatment outcomes. Second, patching duration may be influenced by patient compliance, meaning patients with better compliance had a shorter patching duration. Patient compliance with patching was identified as a key factor for efficacy in treating amblyopia [20] and this could also be applied in treating IXT. Long-term successful outcomes and low recurrence rates have also been associated with good patient compliance with patching for exotropia after surgery [21, 22]. Moreover, older age contributed to treatment success in the current study. This could have also been due to the good compliance of older school-aged patients.

Freeman et al. [7] found patch therapy useful in children aged from 9 months to 5 years to delay surgery and convert exotropia into exophoria or orthophoria. However, evidence highlighting the benefit of patch therapy in children aged 12–35 months is still lacking, with studies showing uncommon deterioration over 6 months among both treated and untreated children, showing a lack of benefit of patch therapy [23]. Given these contradictory findings, watchful waiting was proposed for children aged 12–35 months [24]. Recently, a study demonstrated lower deterioration in the patch therapy group than in the observation group among children aged 3–10 years [25]. Furthermore, Alkahmous et al. [10] reported improved sensory fusion and deviation control among children aged 4–10 years. In this study, although age was not a significant factor after adjustment, the HR for treatment success was increased by 1.25 with an increase of 1 year in age.

Successfully treated patients were followed up for at least 1 year after showing a deviation range of 10 PD or under. They showed average of 12.5 (2–20) PD and 13.18 (4–23) PD of angle decrease in each near and far deviation. In the recurring group, the mean duration after the last day of the normal deviation range was 6.53 months. Although none of the factors identified were found to be statistically significant among the recurring and non-recurring cases, older age seemed to be associated with a reduced risk of recurrence. However, this may be attributed to poor treatment compliance among younger patients. In addition, there was a difference in baseline age between the groups.

Studies addressing the factors associated with patch therapy success are limited. Choi et al. conducted a study with children aged 3–10 years who underwent a fixed 2 h daily patching regimen for 1 year [14] and reported that convergence insufficiency-type exotropia, large exodeviation, and poor distance stereoacuity were factors for improvement after occlusion therapy. The present study showed that patients with a log arcsec of ≤ 1.70 had significantly longer patching duration than patients with a log arcsec of > 1.70, which was similar to the results of the study by Choi et al. [14]. Upon presentation to our clinic, stereoacuity was recorded in only 11 of the 29 patients in the treatment success group, mainly due to the poor cooperation owing to their young age. Therefore, the small sample size should be considered when interpreting the results. Smaller exodeviation was found to be an impacting factor for treatment success in the univariate analysis, with an HR of 0.917 with an increase of 1 PD. While this finding is in contrast with the results of a previous study [14], an accurate comparison cannot be made between these studies because of the differences in study designs. More importantly, this study defined treatment success as ≤ 10 PD, meaning that patients were improved to a point where surgery was no longer needed. In contrast, Choi et al. defined patch responders as a decrease in exodeviation of ≥ 10 PD, improved control scores of ≥ 1, and improved stereoacuity of ≥ 0.6 log arcsec.

This study has some limitations. First, owing to its retrospective design, patching was applied at different hours per day, meaning that each patient received a different patching regimen. Second, patient compliance was not recorded; thus, actual treatment performance could not be accurately estimated. Third, this study only evaluated near stereoacuity which could result in different outcome, making it difficult to compare with studies which had meaningful results on distance stereopsis. Last, 1 year is a relatively short follow-up period to track recurrence in patients.

Nonetheless, this is one of the few studies investigating the potential factors affecting patch therapy success and recurrence. Moreover, this study included patients whose condition was improved with patch therapy and those who were excluded from surgical intervention. Although age was not a significant factor in this study after adjustment, it still showed some association with the treatment success and recurrence. Patch duration was found to be a significant factor, and concerning the relevance between good compliance and older age shown in previous studies [22], good compliance seems to be a contributing factor to treatment success. Our study sample comprised only 59 patients; this was relatively small. With a larger sample size, the statistical analysis of the age factor may have produced different results. Therefore, a larger-scale case-control study is required to determine the key contributing factors and confirm a narrow indication for successful patch therapy outcomes.

## Conclusions

In patch therapy for patients with IXT, treatment success depends on patch duration and adherence to the regimen, with age also playing a significant role.

## Electronic Supplementary Material

Below is the link to the electronic supplementary material.

Supplementary Material 1

Supplementary Material 2

## Data Availability

The datasets used and/or analyzed during the present study are available from the corresponding author upon reasonable request.

## Abbreviations

IXT: Intermittent exotropia
PD: Prism diopter
HR: Hazard ratio
CI: Confidence interval
