# Supplementary material for: Factors influencing the success of patch therapy in patients with intermittent exotropia: a retrospective case-control study
Source: BMC Ophthalmol. 2026 Apr 11;26:176. doi: 10.1186/s12886-026-04774-0 (PMC13077964; doi:10.1186/s12886-026-04774-0)
Supplement: Supplementary file 1 — Supplementary Material 1 [file 12886_2026_4774_MOESM1_ESM.docx]

**Additional Table 1. Demographic and Clinical Features of the Non-recurring and Recurring Treatment Success Groups**

|  | **Non-recurring**  **(n = 16)** | **Recurring**  **(n = 13)** | ***P*-Value** |
| --- | --- | --- | --- |
| Age, in years | 6.75 ± 3.07 (1 – 11) | 4.62 ± 2.29 (1 – 8) | .05 |
| Sex |  |  | .38 |
| Female | 6 (37.5) | 7 (53.85) |  |
| Male | 10 (62.5) | 6 (46.15) |  |
| LogMAR_OD | 0.25 ± 0.28 (0 – 0.9) | 0.31 ± 0.24 (0 – 0.7) | .65^†^ |
| LogMAR_OS | 0.15 ± 0.16 (0 – 0.52) | 0.29 ± 0.25 (0 – 0.7) | .11^†^ |
| SER, D_OD | -0.79 ± 1.41 (-3.25 - +1.5) | -0.17 ± 1.35 (-2.75 - +1.5) | .25 |
| SER, D_OS | -0.40 ± 1.23 (-2.93 - +2.0) | -0.24 ± 1.42 (-2.63 - +1.25) | .75 |
| Angle_Far | 17.31 ± 4.98 (12 – 25) | 19.00 ± 4.97 (12 – 30) | .37^†^ |
| Angle_Near | 20.13 ± 5.82 (12 – 30) | 19.69 ± 4.63 (14 - 30) | .83 |
| Control_Far |  |  | .53 |
| Good | 3 (30) | 0 (0) |  |
| Fair | 3 (30) | 3 (50) |  |
| Poor | 4 (40) | 3 (50) |  |
| Control_Near |  |  | .63^*^ |
| Good | 2 (20) | 0 (0) |  |
| Fair | 4 (40) | 3 (50) |  |
| Poor | 4 (40) | 3 (50) |  |
| Log Arcsec | 1.86 ± 0.58 (1.30 – 3.48) | 1.87 ± 0.38 (1.60 – 2.30) | .98 |
| Patch |  |  | .41^**^ |
| OD | 3 (18.75) | 5 (38.46) |  |
| OS | 4 (25) | 4 (30.77) |  |
| Alternative | 9 (56.25) | 4 (30.77) |  |
| Patch_Time/day (h) | 2.69 ± 1.01 (1 – 4) | 3.31 ± 0.95 (2 – 4) | .10^†^ |
| Patch_Duration (months) | 15.19 ± 18.37 (3 – 74) | 14.54 ± 11.27 (5 – 46) | .91^†^ |
| Glasses prescribed |  |  |  |
| Yes | 9 (64.29) | 6 (75) | > .99 |
| No | 5 (35.71) | 2 (25) |  |

Abbreviations: OD, oculus dexter; OS, oculus sinister; PD, prism diopter; SER, spherical equivalent refraction.

Values are presented as number (%) or means ± standard deviations.

^*^Chi-square test, **Fisher’s exact test, ^†^independent t-test
