# Supplementary material for: Factors influencing the success of patch therapy in patients with intermittent exotropia: a retrospective case-control study
Source: BMC Ophthalmol. 2026 Apr 11;26:176. doi: 10.1186/s12886-026-04774-0 (PMC13077964; doi:10.1186/s12886-026-04774-0)
Supplement: Supplementary file 2 — Supplementary Material 2 [file 12886_2026_4774_MOESM2_ESM.docx]

**Additional Table 2. Logistic Regression Analysis of Clinical Factors of the Non-recurring and Recurring Treatment Success Groups**

| **Parameters** | | **Univariate Analysis**  **OR (95% CI)** | ***P*-Value** |
| --- | --- | --- | --- |
| Sex | Male | ref. |  |
|  | Female | 1.944 (0.439–8.608) | .38 |
| Age, in years | | 0.747 (0.552–1.01) | .06 |
| Angle_Far | | 1.076 (0.92–1.257) | .36 |
| Angle_Near | | 0.984 (0.853–1.135) | .82 |
| LogMAR OD | | 2.219 (0.084–58.742) | .63 |
| LogMAR OS | | 37.402 (0.342–> 999.999) | .13 |
| SE_OD | | 1.415 (0.792–2.527) | .24 |
| SE_OS | | 1.106 (0.616–1.987) | .74 |
| Control_Far | Good | ref. |  |
|  | Fair | > 999.999 (< 0.001–> 999.999) | .98 |
|  | Poor | > 999.999 (< 0.001–> 999.999) | .98 |
| Contro_Near | Good | ref. |  |
|  | Fair | > 999.999 (< 0.001–> 999.999) | .97 |
|  | Poor | > 999.999 (< 0.001–> 999.999) | .97 |
| Log Arcsec | | 1.046 (0.076–14.427) | .97 |
| Patch_Duration/day (h) | | 0.997 (0.950–1.047) | .91 |
| Patch_Time (min) | | 1.923 (0.874–4.231) | .10 |

Abbreviations: OR, odds ratio; CI, confidence interval; OD, oculus dexter, OS, oculus sinister, SER, spherical equivalent refraction.
